# Study Protocol for a Randomized Controlled Trial of Low Intensity Shockwave Therapy for the Treatment of Post-Radical Prostatectomy Erectile Dysfunction: “SHARP-ED TRIAL”

**Published:** 2024-04-08

**Authors:** Francis Petrella, Braian R Ledesma, David Velasquez, Manuel Molina, Russell G Saltzman, Sanoj Punnen, Paul H Chung, Ranjith Ramasamy

**Affiliations:** 1 Desai Sethi Urology Institute, Miller School of Medicine, University of Miami, Miami, Florida, USA;; 2 Department of Urology, Sidney Kimmel Medical College, Thomas Jefferson University, Philadelphia, Pennsylvania, USA

**Keywords:** Clinical trials, Erectile dysfunction, Shockwave therapy, Restorative therapy

## Abstract

**Introduction:**

Erectile Dysfunction (ED) is a common challenge post Radical Prostatectomy (RALP), affecting men’s sexual health after undergoing definitive cancer therapy. Despite employing nerve-sparing techniques, ED remains a prevalent issue in this population. Studies indicate that approximately 70%–85% of men experience varying degrees of ED following RALP. The existing treatment landscape for post-RALP-ED presents limitations, and a discernible knowledge gap persists. To address this, our study aims to investigate the efficacy of Shockwave Therapy (SWT) as a potential intervention for managing ED after RALP.

**Methods:**

This prospective, randomized, sham-controlled clinical trial aims to recruit 189 eligible patients post-RP and assess the effects of SWT. Comprehensive screening, including medical history, physical examinations, and biochemical evaluations, will be conducted to confirm eligibility. The intervention involves utilizing a device to administer focal shockwaves targeted at cavernosal tissue. Safety measures include continuous monitoring for adverse events and rigorous reporting protocols. The primary endpoint assesses changes in participants’ ability to engage in penetrative intercourse from baseline to study completion, while secondary endpoints encompass various measures of erectile function, including questionnaire-based assessments, ultrasound parameters, and clinical outcomes.

**Results:**

Statistical analysis, encompassing ANOVA for continuous variables and Fisher’s exact test for categorical ones, will evaluate demographic characteristics, baseline data, and primary as well as secondary outcomes for statistical significance. Detailed analysis of trends, subgroup comparisons, and treatment effects will provide a comprehensive understanding of the impact of SWT on post-RP ED.

**Conclusion:**

This study protocol represents a rigorous investigation into the potential therapeutic role of SWT in managing post-RP ED. The outcomes from this study aim to contribute valuable insights into the efficacy, safety, and potential improvements in erectile function following SWT, providing significant guidance for future interventions aimed at addressing this challenging condition affecting men’s health and quality of life.

## INTRODUCTION

The surgical procedure of radical prostatectomy is associated with damage to the Cavernous Nerves (CN) and erectile dysfunction. Each year there are approximately 250,000 newly diagnosed cases of Prostate Cancer (PCa) in the United States; overall, it is estimated that 15.3% of all men will be diagnosed with PCa at some point during their lifetime. Radical Prostatectomy (RP) is a common surgical treatment used for localized PCa. Although the long-term oncologic outcome following RP are good, a significant number of patients suffer from post-surgical lower urinary tract complications, including incontinence and ED [1–4]. In most patients, incontinence resolves within 3-months. However, ED may persist for years, and many patients never fully recover erectile function. The prevalence of ED following RALP is a significant concern, with studies indicating a substantial impact on men’s sexual health. Current research suggests that approximately 80% of men may experience varying degrees of ED after undergoing RALP [2], highlighting the high number of men affected by this condition in the post-surgical period. Despite advancements in surgical techniques and the implementation of nerve-sparing approaches, ED remains a prevalent and challenging consequence for a substantial majority of individuals who undergo RALP.

Nerve injury is recognized as a key-factor in the development of post-surgical erectile dysfunction. Even though nerve-sparing techniques are now routinely practiced when performing RP, CN injury, as a result of thermal damage, ischemic injury, mechanically induced nerve stretching and inflammatory effects, remains a major factor in development of ED following surgery. Compounding the acute effects of RP on erectile function, the loss of stimulatory neuronal signals that initiate spontaneous erections results in an overall reduction in oxygenation of erectile tissue, which leads to progressive changes in penile architecture that include induction of smooth muscle apoptosis, fibrosis and deposition of collagen. The effects of long-term ED on penile architecture are such that even when there is CN regeneration, erectile function cannot be fully recovered. Prevention of chronic ED following RP is often considered a race in time between CN regeneration versus irreversible changes in penile architecture.

Accelerating cavernous nerve regeneration has shown potential in improving erectile function outcomes in animal models of radical prostatectomy. Although PDE5 inhibitors represent the first-line oral treatment for ED, their mechanism of action is dependent on the release of nitric oxide from functional CN innervated erectile tissue. Therefore, because of iatrogenic damage to the CN, the vast majority of patients undergoing RP are refractory to treatment of ED by PDE5 inhibitors (PDE5i). In the absence of effective oral treatments for ED, patients that undergo RP are recommended to undergo “penile rehabilitation”. This has a goal of maintaining the erectile tissue oxygenation levels in the acute phase of CN injury to prevent changes in penile architecture, until a time when there is functional CN repair. The strategy commonly utilizes (separately or in combination) intracavernosal injections, vacuum erection devices and low dose PDE5i administration.

The University of Miami “Erectile Preservation Protocol” is used for patients that will undergo a radical prostatectomy. This involves oral PDE5i which treat ED. Patients require additional treatment in order to obtain penetration in roughly 60%–75% of cases [5]. These more invasive treatments involve intracavernosal injections at the time of intercourse. The goal of the study is to have patients involved in penetrative intercourse as rapidly as possible without the use of more invasive treatment options.

The study is aimed at determining the safety and effectiveness of SWT in a patient population soon after? (Maybe even include the number of days to emphasize this is soon after RP) after radical prostatectomy. The rationale behind this is that SWT has been known to bolster angiogenesis and neurogenesis by increasing the levels of vascular endothelial growth factor. The shockwaves are focused onto linear segments that are aimed at the left and right corpora cavernosa and the crura. SWT has previously been shown to improve IIEF scores for organic erectile dysfunction [6]. However, there is a lack of sufficient data on the use of Li-ESWT for penile rehabilitation in the treatment of ED after prostate cancer therapy [7,8]. Existing protocols for SWT are not standardized and involve a limited number of participants with short follow-up periods. Our study aims to evaluate this question using a robust randomized protocol using an adequate number of participants and longitudinal follow up soon after RP.

## MATERIALS AND METHODS

The study adopts a prospective, randomized, sham-controlled clinical trial design to evaluate the safety and efficacy of Shockwave Therapy (SWT) in managing ED post-radical prostatectomy. 189 eligible patients meeting specific inclusion criteria are included and randomly assigned in a 2:1 ratio into either an active treatment group or a sham group. They will be enrolled 6 weeks after their radical prostatectomy to ensure an undetectable PSA. The total duration of the study will be 13 months. This includes pre-treatment screening, 6-months of shockwave therapy sessions, and follow-up at 6-months post treatment.

The comprehensive medical evaluation process includes thorough screening for eligibility, detailed assessment of medical history, comprehensive physical examination, and a battery of blood tests to measure testosterone levels and other relevant biochemical markers. The International Index of Erectile Function (IIEF-EF) questionnaire is employed to assess ED severity and confirm eligibility, while past usage of PDE5 inhibitors is documented.

The SWT intervention utilizes the MoreNova device, directing shockwaves toward specific cavernosal tissue segments. The rationale for utilizing SWT is based on its recognized potential to stimulate angiogenesis and neurogenesis, offering potential in facilitating the recovery of erectile function post-RP.

### Participants

The inclusion criteria are as follows: (i) Be willing and able to provide informed consent; (ii) Be male; (iii) Be ≥ 40 and ≤ 75 years of age (inclusive); (iv) Undergo radical prostatectomy for prostate cancer (nerve sparing or non-nerve sparing without neoadjuvant chemotherapy or radiation therapy.); (v) Be at least six weeks post radical prostatectomy; (vi) Be in a stable sexual relationship for over 3 months prior to enrollment; (vii) Have a serum testosterone level of 300 ng/dL-1000 ng/dL within 1 month prior to enrollment; (viii) Have a HbA1c level ≤ 7% within 3 months prior to enrollment; and (ix) Have undetectable PSA value at the time of enrollment. Patients of all prostate cancer grades/stages and Gleason scores will be included as long as no adjuvant or salvage treatment is needed. Patient drop out will be due to patient preference, adverse effects, cancer recurrence, or need for further cancer treatment.

### Interventions

MoreNova is a Linear Shockwave (LISW) device, which incorporates a shockwave transducer operable to deliver shockwaves to a treatment region confined to a narrow rectangle. Shockwaves generation follows the electromagnetic principle. Linear Shockwaves (LISW), as a treatment for ED has been in evaluation in contemporary medicine, it has been in use for the last ten years. The present study will utilize a device called “MoreNova”, in which shockwaves are focused onto linear segments for improved organ coverage. Shockwaves produced by “MoreNova” are aimed at the left and right corpora cavernosa and the crura. The study is aimed at determining the safety and effectiveness of this new type of LISW in the treatment of ED. The rationale behind this is that LISW has been known to bolster angiogenesis and neurogenesis by increasing the levels of vascular endothelial growth factor [9].

### Endpoints

The primary endpoint includes changes in the participant’s ability to have penetrative intercourse (without the use of intracavernosal injections, and with or without PDE5i use) from baseline to study completion. This information will be collected using the IIEF-EF questionnaire. The secondary endpoints are sexual activity improvement leading to optimal penetration at follow-ups which will be done according to: (i) Percentage of participants who achieve Minimal Clinically Important Differences (MCID) in IIEF score based on their ED severity at baseline. This is measured using the Change International Index of Erectile Function (IIEF-EF) Questionnaire score; (ii) Change in median International Index of Erectile Function–Erectile Function subdomain (IIEF-EF) from baseline to each follow-up time point in active therapy recipients compared to placebo; (iii) Change in median Sexual Encounter Profile questions number 2 and 3 from baseline to each follow-up time point in active therapy recipients compared to placebo; (iv) Change in median Global Assessment Questionnaire (GAQ) score from baseline to each follow-up time point in active therapy recipients compared to placebo; (v) Change in median Erection Hardness Scale (EHS) score from baseline to each follow-up time point in active therapy recipients compared to placebo; (vi) Changes in duplex ultrasound parameters from baseline to study completion in active therapy recipients compared to placebo.

### Procedures involved

**Figure 1:**
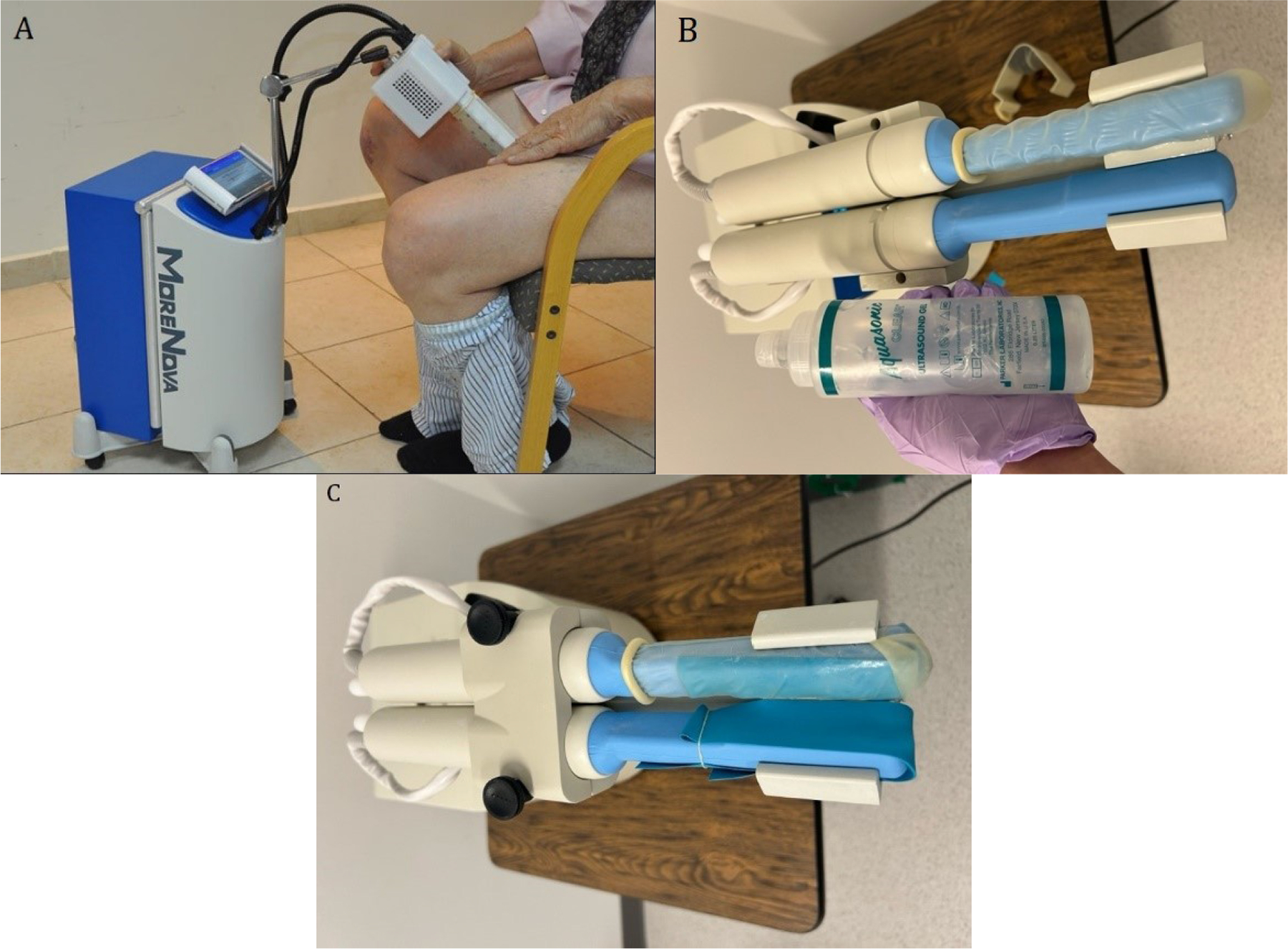
A) Shockwave therapy being administered; B) Shockwave therapy machine for treatment group; C) Shockwave therapy machine for control/ placebo group.

Upon evaluating the inclusion/exclusion criteria, patients will be recruited to the study and randomized into one of the two groups in a 2:1 ratio (randomization will be performed by a computer software maintained by the Desai Sethi Urology Institute). All participants will be instructed to follow the standard post-RP protocol for ED. Treatments will begin 6 weeks after the RP. The treatment sessions last approximately 20 minutes and are performed in an office environment (see below for session and treatment details). Participants randomized to the treatment group will receive one (1) shockwave session once per week for four (4) weeks, followed by one (1) shockwave session once per month (30 ± 7 days) for the next five (5) months. 1,440 shocks of treatment energy will be applied in every session to each treated region (left and right corpora cavernosa and crura) for a total of 12,960 shocks (Figure 1).

Participants randomized to the sham (control) group will have the same number of sessions, and the machine will be set to deliver 1,440 shock treatments, but a barrier will be placed around the shockwave probe to ensure that no shockwaves are delivered.

### Follow-up interventions

Follow-up visit will be conducted at 6 months after the last treatment session and shall include: (i) Questionnaires to measure the ability to have penetrative intercourse (IIEF-EF, GAQ, SEP, and EHS); (ii) Recording adverse events; and (iii) Penile Doppler Ultrasound (Table 1).

### Safety

Any adverse event (penile hematoma or penile fracture) and eventual complication must be recorded at any time during the treatments and the follow up visits, and throughout the entire study duration. Patients will be instructed to alert the study investigator by telephone of any side effects occurring in the period after the treatment and until the study ends. The investigators will conduct continuous reviews of the data and subject safety, keeping track of the number of subjects, significant toxicities in accordance with the protocol and observed responses, which will be discussed at research committee meetings. All grade 3–5 adverse events (CTCAE v4.0), regardless of association with the SWT, will be entered into study database and reviewed at research committee meetings. In addition, all adverse reactions considered “serious” will be entered into the research database and reviewed by the Surgeon monitor on an ongoing basis.

### Sample size calculation

In our research protocol, we aim to assess the effectiveness of shockwave therapy on patients’ ability to engage in penetrative intercourse after a one-year period. Our initial observations revealed that 20% of patients achieved this without therapy, whereas an aim of 40% will achieve it with shockwave therapy. To design our study with statistical rigor, we utilized an allocation ratio of 2:1 (control:shockwave), and factored in a power of 80% and a significance level of 5%. Employing calculated Z-values for α and β, we derived an estimated sample size requirement of approximately 189 participants. The overall sample size was determined to be 189 patients, with 63 patients in the control group and 126 in the active treatment group.

### Data analysis

The average and standard deviation of all relevant variables, including demographic and baseline characteristics, primary and secondary outcomes will be calculated. IIEF-EF and EHS scores will be analyzed using ANOVA with repeated measures throughout the study, in order to compare the trends between the treatment and sham group. GAQ, SEP and success rates will be analyzed and compared between the groups according to Fisher’s exact test in each of the endpoints. The statistical significance will be set at P<0.05.

Demographic characteristics such as age and ED duration will be compared between treatment group and sham group using student’s T-Test. Other demographic characteristics, such as medical background and risk factors will be compared between these groups using Fisher’s Exact Test.

### Trial registration

IRB: 20231090

Clinicaltrials[dot]gov identifier number: NCT06152146

### Trial status

Pending IRB approval

## RESULTS AND DISCUSSION

The challenge of ED persists as a prevalent issue impacting men’s sexual health. It’s not merely an age-related concern; rather, it affects a substantial percentage of men across various age groups. This study delves into understanding and addressing ED, specifically post- RP, and a common complication despite advancements in surgical techniques. This clinical trial will study a patient population that has been excluded in most previous shockwave therapy trials while representing a large portion of patients with erectile dysfunction.

The complexity of achieving penile erections involves a series of intricate physiological processes. Disruptions in these mechanisms, especially post-RP due to CN injury, lead to persistent ED. This presents a significant hurdle in the post-prostatectomy recovery phase and adversely affects the quality of life for these individuals.

The introduction of SWT presents a hopeful avenue in managing post-RP ED. The rationale behind using SWT lies in its potential to trigger angiogenesis and neurogenesis, offering a means to counteract the adverse effects of reduced oxygenation and structural changes in penile tissue following RP. This treatment has shown potential in other medical domains, hinting at its regenerative potential and effectiveness.

The use of SWT has shown potential in several studies. Initially it was demonstrated that SWT has a direct role of angiogenesis in rat models with pelvic nerve injuries [10]. This was confirmed in prostatectomy animal models [11]. There are numerous studies on humans for which metanalyses have demonstrated positive outcomes in patients with organic erectile dysfunction [7,12]. The limitation of these studies is that patients with a radical prostatectomy are usually excluded. Matthew et al, have recently reviewed the literature on the role of SWT in the post prostatectomy population and determined that more studies are needed with longer follow-ups and larger patient populations [7]. A current study out of Thomas Jefferson University Hospital is taking place under the leadership of Dr. Paul Chung to study the use of SWT after RALP. Our studies differ in inclusion criteria, study duration and primary endpoint.

The study’s design, adopting a prospective, randomized, and sham-controlled approach, underscores its robustness in evaluating SWT’s efficacy. Rigorous safety measures, including comprehensive screening and continuous monitoring for adverse events, ensure participant safety and data reliability. Assessing changes in participants’ ability to engage in penetrative intercourse serves as the primary endpoint. This differs compared to most studies assuming that symptom score scales are more appropriate. We believe that the role of SWT would be to decrease the ratio of patients requiring more invasive treatments in order to have penetrative intercourse. Supplementary endpoints, such as questionnaire-based evaluations and ultrasound parameters, also provide a holistic understanding of SWT’s impact on post-RP ED.

While the study aims to address this critical issue, inherent limitations, such as potential biases and challenges in blinding due to the intervention’s nature, exist. Nevertheless, positive outcomes could prepare for incorporating SWT into the management strategy for post-RP ED, potentially offering a solution for affected individuals. In essence, this study represents a significant stride in investigating an innovative therapeutic approach for a persistent challenge in men’s health. The outcomes will not only contribute to the scientific understanding of post-RP ED but also potentially offer a practical solution to improve the lives of those affected.

## CONCLUSION

In summary, this study explores Shockwave Therapy (SWT) as a potential intervention for managing Erectile Dysfunction (ED) Post-Radical Prostatectomy (RP). By rigorously evaluating SWT’s efficacy and safety through a prospective, randomized, sham-controlled clinical trial, this study aims to offer insights into addressing persistent ED after RP. Positive outcomes could potentially introduce SWT as an effective therapeutic option, benefiting individuals affected by post-RP ED and improving their quality of life.

## Figures and Tables

**Table 1:** The details of the events and procedures participants will undergo throughout the trial.

Schedule of events	Screening	Weekly treatment					Monthly treatment				Follow-up
Visit	Visit 1	Visit 2	Visit 3	Visit 4	Visit 5	Visit 6	Visit 7	Visit 8	Visit 9	Visit 10	Visit 11
Time point	Screening	Week 1	Week 2	Week 3	Week 4	Week 8	Week 12	Week 16	Week 20	Week 24	Month 12
Activity window	0–30 days pre	± 3 days	± 3 days	± 3 days	± 3 days	± 3 days	± 7 days	± 7 days	± 7 days	± 7 days	± 30 days
Informed consent	•										
Medical and urological history	•										
Demographics	•										
Physical examination	•										
Penile Doppler ultrasound	•										•
Blood Test: CBC, HbA1c, PSA and Testosterone	•										
Questionnaires: IIEF-EF, GAQ, SEP, EHS	•	•	•	•	•	•	•	•	•	•	•
Verification of eligibility	•										
Randomization		Group A: Treatment Group B: Sham									
Administration of therapy		•	•	•	•	•	•	•	•	•	
Review concomitant medication		•	•	•	•	•	•	•	•	•	
Review adverse events		•	•	•	•	•	•	•	•	•	•

## Data Availability

Data supporting the findings of this study are available from the corresponding author, RR, upon reasonable request.

## ABBREVIATION

ED: Erectile Dysfunction
AE: Adverse Event
EHS: Erection Hardness Score
GAQ: Global Assessment Questions
IIEF-EF: The International Index of Erectile Function–Erectile Function
LISW: Low Intensity Shock Wave
PDE5i: Phosphodiesterase type 5 inhibitor
SEP: Sexual Encounter Profile

## References

[1] Melman A, Gingell JC. The epidemiology and pathophysiology of erectile dysfunction. J Urol. 1999;161(1):5–11. 10037356

[2] Emanu JC, Avildsen IK, Nelson CJ. Erectile dysfunction after radical prostatectomy: Prevalence, medical treatments, and psychosocial interventions. Curr Opin Support Palliat Care. 2016;10(1):102–107. 26808052 10.1097/SPC.0000000000000195 PMC5005072

[3] Ladegaard PB, Mortensen J, Skov-Jeppesen SM, Lund L. Erectile dysfunction a prospective randomized placebo-controlled study evaluating the effect of low-intensity extracorporeal shockwave therapy (LI-ESWT) in men with erectile dysfunction following radical prostatectomy. Sex Med. 2021;9(3):100338. 33789173 10.1016/j.esxm.2021.100338 PMC8240152

[4] Kikuchi Y, Ito K, Ito Y, Shiroto T, Tsuburaya R, Aizawa K, Double-blind and placebo-controlled study of the effectiveness and safety of extracorporeal cardiac shock wave therapy for severe angina pectoris. Circ J 2010;74(3):589–591. 20134096 10.1253/circj.cj-09-1028

[5] McCullough AR. Prevention and management of erectile dysfunction following radical prostatectomy. Urol Clin North Am. 2001;28(3):613–627. 11590817 10.1016/s0094-0143(05)70166-x

[6] Liu PY, Swerdloff RS, Christenson PD, Handelsman DJ, Wang C. Rate, extent, and modifiers of spermatogenic recovery after hormonal male contraception: An integrated analysis. The Lancet. 2006;367(9520):1412–1420. 10.1016/S0140-6736(06)68614-5 16650651

[7] Matthew AN, Rogers DE, Grob G, Blottner M, Kodama S, Krzastek SC. The use of low-intensity extracorporeal shockwave therapy in management of erectile dysfunction following prostate cancer treatment: A review of the current literature. Transl Androl Urol. 2023;12(6):1023. 37426598 10.21037/tau-22-791 PMC10323450

[8] Sighinolfi MC, Eissa A, Bellorofonte C, Mofferdin A, Eldeeb M, Assumma S, Low-intensity extracorporeal shockwave therapy for the management of postprostatectomy erectile dysfunction: A systematic review of the literature. Eur Urol Open Sci. 2022;43:45–53. 35928730 10.1016/j.euros.2022.07.003 PMC9344341

[9] Peng D, Tan Y, Reed-Maldonado AB, Lin G, Lue TF. Molecular mechanism of action of low-intensity extracorporeal shockwave therapy for regenerating penile and peripheral nerves. Turk J Urol. 2020;33:35. 10.5152/tud.2020.20419 33052844

[10] Li H, Matheu MP, Sun F, Wang L, Sanford MT, Ning H, Low-energy shock wave therapy ameliorates erectile dysfunction in a pelvic neurovascular injuries rat model. J Sex Med. 2016;13(1):22–32. 26755082 10.1016/j.jsxm.2015.11.008

[11] Jeon SH, Shrestha KR, Kim RY, Jung AR, Park YH, Kwon O, Combination therapy using human adipose-derived stem cells on the cavernous nerve and low-energy shockwaves on the corpus cavernosum in a rat model of post-prostatectomy erectile dysfunction. Urology. 2016;88:2261–2269. 10.1016/j.urology.2015.10.021 26522972

[12] Clavijo RI, Kohn TP, Kohn JR, Ramasamy R. Effects of low-intensity extracorporeal shockwave therapy on erectile dysfunction: A systematic review and meta-analysis. J Sex Med. 2017;14(1):27–35. 27986492 10.1016/j.jsxm.2016.11.001

